# Association of peripheral leukocyte telomere length and its variation with pancreatic cancer and colorectal cancer risk in Chinese population

**DOI:** 10.18632/oncotarget.9536

**Published:** 2016-05-21

**Authors:** Rui Zhang, Jian Zhao, Jian Xu, Fang Liu

**Affiliations:** ^1^ Department of Colorectal surgery, Liaoning cancer hospital & institute, Shenyang, Liaoning Province, 110042, P.R. China

**Keywords:** cancer, telomere length, LTL, TLV, risk

## Abstract

There is increasing evidence supporting the role of telomeres in cancer pathogenesis. However, limited studies have investigated the association between telomere length features and risk of pancreatic cancer and colorectal cancer (CRC), and little was conducted in Asians. To help clarify this issue, We measured relative peripheral leukocytes telomere length (LTL) and telomere length variation (TLV) in a prospective study of 900 pancreatic cancer cases, 300 CRC cases, and 900 controls. Both subjects with longer LTL (quartile 4: adjusted OR=1.51, 95% CI: 1.14-1.99, P=0.004) and shorter LTL (quartile 1: adjusted OR=3.12, 95% CI: 1.89-5.14, P=8.50×10^−6^) showed increased risk of pancreatic cancer. A linear increased risk was detected For TLV (adjusted OR=1.60, 95% CI: 1.14-2.24, P=0.006). We also identified significant interaction for relative LTL, TLV on pancreatic cancer risk (P interaction =0.009). Significant relationship between shorter RTL and increased CRC risk were also detected. This findings provide insights into telomere dynamics and highlight the complex relationship between relative LTL, TLV and cancer risk.

## INTRODUCTION

The incidence of pancreatic cancer and colorectal cancer (CRC) has been increasing for several decades in Chinese population. Pancreatic cancer ranks the fifth leading cause of worldwide cancer death and the fourth leading cause of cancer mortality in the U.S, with an estimated new cases of 48,960 in 2015, and over 40,560 were estimated to die from the disease, while CRC ranks the fourth most common cause of cancer death [1–3]. According to a report of National Office for Cancer Prevention and Control of China, the prevalence of pancreatic cancer has risen up to 17.8 / 1,000 males, and 13.7 / 1,000 females [4]. It is likely that a lack of effective screening methods to detect early disease contributes to its low 5-year survival rate, as few biomarkers have been identified to detect pancreatic cancer and CRC [5–7]. This makes pancreatic cancer the most deadly cancer worldwide, with a 5-year relative survival of less than 5% [8–10]. Although many risk factors includes smoking, alcohol consumption, preexisting diabetes mellitus, obesity, and family history of cancer has been established, these factors, which can provide only a very weak prediction of an individual's pancreas cancer and CRC risk, can only account for a modest proportion of pancreatic cancer and CRC occurrences [11–13].

Telomere length and telomere erosion has been strongly implicated in the molecular biology of many cancers, including pancreatic cancer and CRC [14–17]. When telomere lengths become critically short, the process of cell senescence is initiated, resulting in cell-cycle arrest or apoptosis in normal cells [18–21]. Epidemiological studies have also shown that peripheral leukocytes telomere length (LTL) correlates with the incidence of major chronic diseases [14, 22–26]. LTL has become a biomarker of biological ageing and risk of age-related diseases, like cancers, cardiovascular disease and diabetes [26, 27]. So far, only three studies have investigated circulating LTL and risk of pancreatic cancer [12, 28, 29], while none of them were conducted in Asians. Additionally, recent studies also showed that telomere length variation (TLV) across all chromosomal ends, which could increase chromosome instability and poor clinical outcome, contributed to the risk of bladder cancer [30]. To further clarify the association between LTL, TLV and pancreatic cancer and CRC development, we performed this nested case-control study to examine the degree of association between LTL, TLV and risk of pancreatic cancer and CRC.

## RESULTS

### Characteristics of study population

We measured the LTL in 900 pancreatic cancer cases, 300 CRC cases and in 900 matched control subjects recruited in the context of the prospective CCPSAPP study. The distribution of relevant baseline characteristics of cases and controls is shown in Table 1. There were no significant differences in the distributions of age, gender, BMI, and infection of diabetes. The pancreatic cancer cases were significantly more likely than the controls to be smokers, drinkers, and patients of hypertension (All P value <0.05). Table 2 presents the overall case–control comparison of telomere features and stratified comparisons by age, gender. The pancreatic cases have significantly larger TLV, compared with the healthy controls. Age was inversely related to LTL, among the cases as well as among the healthy controls. While age was positively related to TLV. With respective to gender, women are likely have longer relative LTL and smaller TLV.

**Table 1:** Baseline characteristics of pancreatic cancer cases and controls

Variables	Cases (N=900)	Controls (N=900)	*P* value
Age (years)	54.0±4.11	54.2±4.19	0.307
BMI (kg/m^2^)	26.1±3.1	25.9±2.9	0.158
Gender			
Male	409 (45.4%)	402 (44.7%)	0.740
Female	491 (44.6%)	498 (45.3%)	
Ever smokers	317 (35.2%)	264 (29.3%)	**0.008**
Ever drinkers	371 (41.2%)	295 (32.8%)	**P<0.001**
Diabetes			
Yes	387 (43.0%)	354 (39.3%)	0.114
No	513 (57.0%)	546 (60.7%)	
Hypertension			
Yes	537 (59.7%)	403 (44.8%)	**P<0.001**
No	363 (40.3%)	497 (55.2%)	

**Table 2:** Case–control comparison of telomere features

Telomere features	Cases mean (SD)	Controls mean (SD)	P value
All subjects, N	900	900	
Relative LTL	1.49 (0.21)	1.51 (0.23)	0.054
TLV	60.1 (9.8)	56.2 (10.1)	P <0.001
Age>60, N	398	410	
Relative LTL	1.45 (0.35)	1.47 (0.31)	0.858
TLV	63.2 (11.4)	59.8 (10.9)	P <0.001
Age≤60, N	502	490	
Relative LTL	1.52 (0.25)	1.54 (0.27)	0.250
TLV	58.5 (9.1)	54.3 (9.5)	P <0.001
Male	409	402	
Relative LTL	1.48 (0.31)	1.50 (0.37)	0.327
TLV	60.9 (10.2)	56.7 (11.2)	P <0.001
Female	491	498	
Relative LTL	1.50 (0.31)	1.52 (0.27)	0.280
TLV	59.2 (8.9)	56.1 (9.0)	P <0.001

### Association between relative LTL, TLV and risk of pancreatic cancer

Unconditional logistic regression analysis was used to evaluate the association between relative LTL, TLV and pancreatic cancer risk. As shown in Table 3, significant trend was detected for associations between relative LTL, TLV and risk of pancreatic cancer (P<0.01). For relative LTL, the third quartile was set as the reference. Both subjects with longer LTL (quartile 4: adjusted OR=1.51, 95% CI: 1.14-1.99, P=0.004) and longer LTL (quartile 1: adjusted OR=3.12, 95% CI: 1.89-5.14, P=8.50×10^−6^) showed increased risk of pancreatic cancer. When analyzed as a continuous variable, a significantly increased risk was identified for short relative LTL (adjusted OR=1.50, 95% CI: 1.12-2.01, P=0.007). For TLV, a linear increased risk was detected (adjusted OR=1.60, 95% CI: 1.14-2.24, P=0.006). Compared with quartile 1, both quartile 3 (adjusted OR=2.52, 95% CI: 1.27-4.99, P=0.008) and quartile 4 (adjusted OR=4.27, 95% CI: 1.87-9.75, P=5.67×10^−4^) showed statistically significant trend.

**Table 3:** Associations between relative LTL, TLV and pancreatic cancer risk

Variables	Model A ^1^		Model B ^2^	
	OR (95% CI)	*P*	OR (95% CI)	*P*
**Relative LTL**				
Quartile 4	1.49 (1.13–1.97)	**0.005**	1.51 (1.14-1.99)	**0.004**
Quartile 3	Reference		Reference	
Quartile 2	1.29 (0.98-1.71)	0.073	1.30 (0.98-1.72)	0.069
Quartile 1	3.10 (1.84-5.21)	**2.00×10^−5^**	3.12 (1.89-5.14)	**8.50×10^−6^**
Continuous	1.49 (1.11-2.00)	**0.008**	1.50 (1.12-2.01)	**0.007**
**TLV**				
Quartile 1	Reference		Reference	
Quartile 2	1.80 (0.98-3.32)	0.060	1.79 (0.96-3.32)	0.065
Quartile 3	2.56 (1.25-5.23)	**0.010**	2.52 (1.27-4.99)	**0.008**
Quartile 4	4.31 (1.71-10.89)	**2.01×10^−3^**	4.27 (1.87-9.75)	**5.67×10^−4^**
Continuous	1.65 (1.13-2.15)	**0.007**	1.60 (1.14-2.24)	**0.006**

1 Crude ORs.

2 Adjusted for age, gender, smoking status, drinking status, hypertension, body mass index (BMI) and diabetes.

### Joint effects of relative LTL, TLV on pancreatic cancer and CRC risk

To assess if combinations of relative LTL and TLV increased the risk stratification for pancreatic cancer and CRC, we used the median value of control subjects as cutoff points to create comparison groups. As shown in Table 4, significant interaction was detected for relative LTL, TLV on pancreatic cancer risk (P _interaction_ =0.009). Compared to subjects with longer LTL and smaller TLV, subjects carrying shorter LTL and larger TLV exhibited a 1.38-fold increased risk of pancreatic cancer (OR = 1.38; 95%CI, 1.04-1.82; P =0.023). However, we didn't find any significant interaction for CRC risk (data not shown).

**Table 4:** Joint effects of relative LTL, TLV on pancreatic cancer risk

Variables	Relative LTL					
	Long			Short		
	Case	Control	OR(95% CI)*	Case	Control	OR(95% CI)*
**TLV**						
short	167	200	1.00 (reference)	238	250	1.14 (0.87-1.49)
long	261	247	1.26 (0.97-1.65)	234	203	**1.38 (1.04-1.82)**
	***P* for interaction = 0.009**					

### Association between relative LTL and risk of colorectal cancer

To enhance our founding, we also replicated the association in a case-control study of colorectal cancer. As shown in Table 5, significant relationship between shorter RTL and increased CRC risk were detected (adjusted OR=1.27, 95% CI: 1.07-1.51, P=0.007).

**Table 5:** Association between telomere length and colorectal cancer risk

Telomere length	Cases	Controls	Adjusted OR^1^	P value
3rd tertile	75	300	1.00 (reference)	
2nd tertile	101	300	1.34 (0.96-1.89)	0.085
1^st^ tertile	124	300	1.65 (1.19-2.29)	**0.002**
Per tertile			1.27 (1.07-1.51)	**0.007**

1 adjusted by age, sex, smoking status and alcohol use.

## DISCUSSION

In this study, we conducted a nested case–control study in a Chinese population and evaluated the relationships between relative LTL, TLV and risk of pancreatic cancer and CRC. Our results showed that either short or extreme long telomere length were associated with an increased risk of pancreatic cancer. TLV was linearly associated with increased risk of pancreatic cancer. We also found significant interaction for relative LTL, TLV on pancreatic cancer risk. We also found that shorter RTL was associated with increased CRC risk. These findings provide novel epidemiological evidence for the effect of telomeres on the development of pancreatic cancer and CRC. To the best of our knowledge, this study should be the first study which aims to evaluate the association of telomere features and risk of pancreatic cancer in Asians.

Telomere length, a putative marker of biological age that have a key role in various cellular processes such as control of chromosomal stability, regulation of cell growth, and the proper segregation of chromosomes to daughter cells, has been identified to be associated with cancer development for a long time [15, 31–36]. Previous studies have produced inconsistent results and been hindered by being based on measurements from telomeres that were already exposed to cancer and treatments [37]. In 1997, Kobitsu et al first explore the relationship between shortened telomere length and increased telomerase activity in hamster pancreatic duct adenocarcinomas and cell lines [38]. van Heek et al also reported that telomere shortening was nearly universal in pancreatic intraepithelial neoplasia [39]. In this context, only three studies, including one case-control study which found short telomeres and extremely long telomeres in peripheral blood are associated with an increased risk for pancreatic cancer [12], and two nested case-control studies which presented the inconsistent results [28, 29]. However, none of the studies was conducted in Asians, not to say Chinese. Thus, to provide the evidence for the association between the telomere features and risk of pancreatic cancer in Asians, we conducted this study.

Telomeres are the end caps of chromosomes and are composed of telomeric proteins and hexamer repeats of DNA (TTAGGG) that serve to protect the integrity of the DNA sequence during cell division [40]. Our findings suggested that either short or extreme long telomere length were associated with an increased risk of pancreatic cancer. This was similar to the conclusion of Skinner et al [12], although the limited sample size and the study design of case-control study. Lynch et al [22] identified that longer telomere length was significantly associated with increased pancreatic cancer risk (continuous OR =1.26, 95% CI 5 1.09–1.46), while Campa's results don't support LTL as a uniform and strong predictor of pancreatic cancer [26]. One possible explanation for the inconsistent results should be the limited sample size (only 193 pancreatic cancer cases in Lynch's study and 331 cases in Campa's study). In current study, we also included TLV, which represents the heterogeneous telomere length across all chromosomal ends, as a candidate telomere feature. Consistent with our findings, Wang eT al [38] found TLV was strongly associated with an increased bladder cancer risk in Egyptian and the association was modulated by age. TLV measures the combined effects of very short and excessively long telomeres and represents a novel aspect of telomere function and might be a better biomarker for cancer risk.

In conclusion, this is the first study demonstrating that subjects with either short or extreme long telomeres may have an increased risk of pancreatic cancer in Asian population. Strengths in this study includes the prospective study design, the largest sample size, and the usage of TLV measure. The findings provided valuable clues for better understanding the underlying contribution of telomeres to carcinogenesis.

## MATERIALS AND METHODS

### Study population

The current study is part of the Cancer Prevention Study of Adolescent Population in Liaoning Province, China, since June 2010. This is a relatively large cohort of individuals who visit us for an annual check-up. Epidemiological survey was conducted using a structured questionnaire. Blood samples were collected at baseline recruitment and fractionated into serum, plasma, erythrocytes, and buffy coat from which DNA was extracted. Written informed consent for participation was obtained. Until Jan 2015, a total of 1,200 incident cases of pancreatic cancer and CRC, which were histologically proven and available for Relative LTL assessment, were recruited. Patients who had a prior history of other cancers were excluded. For each case, the pathology reports were double-reviewed by either one of the two study pathologists. Matched controls were randomly selected from the cohort who were alive and free of cancer at the time the case was diagnosed.

### Measurement of relative LTL and TLV

Genomic DNA was isolated from peripheral blood leukocytes using the QIAmp 96-spin blood protocol (Qiagen, Chatsworth, CA, USA). Relative LTL was determined using the real-time quantitative polymerase chain reaction (PCR) method. Using an Applied Biosystems 7900 Sequence Detection System, each sample was assayed in duplicate in separate 384-well plates. Relative LTL in genomic DNA was determined using the telomere (T) copy number to single-copy (S) b-globin (HGB) gene copy number. T/S in this study is a relative and dimensionless value, which is proportional to the average telomere length per cell. For TLV measurement, first, blood lymphocyte cultures were set up following the protocol described previously [41]. Then, telomere length at each of the chromosomal ends was measured by telomere quantitative fluorescent in situ hybridization (TQ-FISH). After TQ-FISH, cells were analyzed using an epifluorescence microscope equipped with a charge-coupled device camera. Case and control samples were analyzed together in each batch. Laboratory personnel were blinded to participant characteristics and all assays were processed in triplicates.

### Statistical analyses

All analyses were performed using SAS software (Version 9.3, SAS Institute Inc.). All statistical tests were two-sided, and P < 0.05 was considered statistically significant. The difference in the distribution of host characteristics between subjects groups was assessed by the Student's t-test for continuous variables, and the Pearson chi-square test for categorical variables. LTL measurements were log-transformed to obtain a variable with an approximately normal distribution. Relative LTL was categorized using the quartile in controls as cutoff points. Unconditional multivariate logistic regression was used to calculate odds ratio (ORs) and 95% confidence intervals (CI) for the he strength of association between telomere features and the risk of pancreatic cancer and CRC, adjusting for age, gender, smoking status, drinking status, hypertension, body mass index (BMI) and diabetes (where appropriate).
